# Intimate partner violence around the time of pregnancy and postpartum depression: The experience of women of Bangladesh

**DOI:** 10.1371/journal.pone.0176211

**Published:** 2017-05-04

**Authors:** Md. Jahirul Islam, Lisa Broidy, Kathleen Baird, Paul Mazerolle

**Affiliations:** 1 School of Criminology and Criminal Justice, Griffith University, Brisbane, Queensland, Australia; 2 Ministry of Planning, Bangladesh Planning Commission, Sher-e-Bangla Nagar, Dhaka, Bangladesh; 3 Department of Sociology, 1 University of New Mexico, Albuquerque, New Mexico, United States of America; 4 School of Nursing and Midwifery, Menzies Health Institute Queensland, Griffith University, Brisbane, Queensland, Australia; Indiana University, UNITED STATES

## Abstract

**Background and objectives:**

Intimate partner violence (IPV) around the time of pregnancy is a serious public health concern and is known to have an adverse effect on perinatal mental health. In order to craft appropriate and effective interventions, it is important to understand how the association between IPV and postpartum depression (PPD) may differ as a function of the type and timing of IPV victimization. Here we evaluate the influence of physical, sexual and psychological IPV before, during and after pregnancy on PPD.

**Methods:**

Cross-sectional survey data was collected between October 2015 and January 2016 in the Chandpur District of Bangladesh from 426 new mothers, aged 15–49 years, who were in the first six months postpartum. Multivariate logistic regression models were used to estimate the association between IPV and PPD, adjusted for socio-demographic, reproductive and psychosocial confounding factors.

**Results:**

Approximately 35.2% of women experienced PPD within the first six months following childbirth. Controlling for confounders, the odds of PPD was significantly greater among women who reported exposure to physical (AOR: 1.79, 95% CI [1.25, 3.43]), sexual (AOR: 2.25, 95% CI [1.14, 4.45]) or psychological (AOR: 6.92, 95% CI [1.71, 28.04]) IPV during pregnancy as opposed to those who did not. However, both before and after pregnancy, only physical IPV evidences a direct effect on PPD. Results highlight the mental health consequences of IPV for women of Bangladesh, as well as the influence of timing and type of IPV on PPD outcomes.

**Conclusions and implications:**

The findings confirm that exposure to IPV significantly increases the odds of PPD. The association is particularly strong for physical IPV during all periods and psychological IPV during pregnancy. Results reinforce the need to conduct routine screening during pregnancy to identify women with a history of IPV who may at risk for PPD and to offer them necessary support.

## Introduction

Culturally, pregnancy is often viewed as a time of happiness and expectancy in women’s lives, with the welcoming of the next generation and growing anticipation of the joys a new child will bring to the family. At the same time, pregnancy can also be a stressful and anxiety-provoking life event [1], and many women experience perinatal mental health problems during this period [2]. Studies from high-income countries have revealed that the prevalence of depressive symptoms during pregnancy is equal to or even higher than that of the postpartum period [3–5]. However, postpartum depression (PPD) has become a matter of concern, affecting approximately 10–20% of new mothers worldwide [6–8]. PPD occurs within four weeks of childbirth [6] with symptoms that can include sadness, anxiety, loss of interest or pleasure in daily activities, constant fatigue, poor concentration, disturbed sleep or appetite, feelings of guilt or low self-worth, social withdrawal and excessive crying [9, 10]. According to the World Health Organization (WHO), depression will be the second leading cause of the disease burden for women in high, middle- and low-income countries by 2020 and is expected to move into first place by 2030 [10].

PPD has been recognized as a significant global public health concern due to its profound health consequences for both mothers and their families [2, 11]. PPD is associated with a host of negative maternal, infant, and family outcomes. These include impaired mother-infant interactions [12–14], parenting stress [15], maternal deaths due to suicide [2, 6] and paternal depression [2]. Not surprisingly, a mother’s PPD also affects her child. Infants whose mothers experience PPD are at risk for malnutrition [16, 17], poor growth [18, 19], illness [17] and even mortality [20–22]. Moreover, as they develop they often exhibit delays in reaching key milestones of cognitive and emotional functioning [6, 22–24]. This likely stems from the fact that depressed mothers express more negative emotions, and this is associated with more limited vocal and visual communications with their infants, fewer positive facial emotions and verbal expression and less physical affection [25]. Further, newborns of depressed mothers demonstrate more perinatal problems, including a decreased response to stimulation evidenced by fewer smiles, less playfulness, more irritability and fussiness [22]. PPD has also been associated with early termination of exclusive breastfeeding [26, 27]. With such compelling evidence, it is important to identify the risk factors for PPD. While some of these risks are well established [6, 10, 28], recent evidence suggests that a history of intimate partner violence (IPV) is among the most notable risks [29] and thus worthy of further scrutiny.

IPV includes acts of physical, sexual and psychological coercion along with controlling behaviors against women by a current or former intimate partner [30, 31]. One of the most concerning elements of IPV is that pregnancy does not offer women protection against IPV [32–34]. A recent meta-analysis established the prevalence of IPV during pregnancy to be anywhere from 4.8 to 63.4% [32], depending on the definition, assessment tools and population. The postpartum period is also considered a time of increased risk of IPV for new mothers [35, 36], with prevalence rates ranging from 2–25% [37]. During pregnancy, IPV may increase in more frequently [38] and more severely [39]. A growing number of studies have demonstrated that IPV victimization around the time of pregnancy is associated with postpartum mental health problems [40–47].

Although our understanding regarding the links between IPV and PPD is progressing, notable gaps remain. Until recently the majority of research has focussed primarily on physical IPV [43, 48], and therefore we know very little about how different forms of IPV might impact PPD [43]. Moreover, few studies have explored whether the timing of IPV around pregnancy affects the likelihood of a mother developing PPD symptoms. Recent research suggests that the odds of PPD can change depending on whether IPV occurs before, during or after pregnancy [40, 45, 49, 50]. IPV can start prior to pregnancy; continue during pregnancy and postpartum period. Alternatively, IPV may commence during pregnancy and continue in the postpartum period or commence in the postpartum period only. Martin et al. (2006) found that women who experienced physical or sexual IPV before or during pregnancy had higher levels of depressive symptoms compared with non-victims, whereas experiencing psychological IPV not before but during pregnancy was associated with PPD. At this present time, we do not know any studies from South Asia, including Bangladesh that has specifically examined the impact of all types of IPV victimization before, during and after pregnancy on PPD.

Whilst the literature on IPV in Southeast Asia is growing, it still remains limited and there are few scholarships examining the association between IPV and PPD for women of Bangladesh. To help build this important knowledge base, we assess how the association between IPV and PPD changes as a function of the type and timing of IPV in a population-based sample of new mothers in Bangladesh. The aim of this study is to examine whether:1) recent exposure (occurred after childbirth) to physical, psychological and sexual IPV is associated with PPD in the first six months after childbirth; and 2) prior exposure (occurred before and during pregnancy) to physical, psychological and sexual IPV is also associated with PPD. Understanding the association between PPD outcomes and exposure to different forms of IPV during different periods has clinical implications regarding early detection and targeted preventative measures around the time of pregnancy to support at-risk women.

## Materials and methods

### Setting and participants

A cross-sectional survey was conducted from October 2015 to January 2016 in two sub-districts of the Chandpur district of Bangladesh. New mothers who visited vaccination centers to receive their baby’s vaccinations were the target population. Married women between 15–49 years of age currently living with their husbands for the last two years, and who had at least one child aged six months or younger were eligible for the study. These criteria were used to determine the women’s experience of IPV from their current husband. A multistage random sampling method was adopted to identify vaccination centers from which to draw 426 estimated subjects. Interviewers approached 453 postpartum mothers to reach the desired sample size, yielding a response rate of 94%.

The data collection procedure has been described in detail previously in [27, 51]. Face-to-face structured interviews were conducted in a safe and private room with eligible mothers who agreed to participate in the study. In lieu of self-response schedules, closed-form interviews were conducted due to the relatively low level of literacy among the women. The interviews were conducted by two local female interviewers, with experience and knowledge in sociology, anthropology and quantitative data collection procedures. Each participant received a monetary ‘thank-you gift’ (500BDT ~ 6.50 USD) to compensate for their time. At the end of the interview, each participant was offered a brochure detailing community resources on IPV and mental health, for example, helpline, phone numbers, legal services, which they could access free of charge.

Participation was entirely voluntary and confidential and did not affect receiving health care in any way. Women who reported an experience of IPV were offered primary counselling services and referrals to local social and psychological services. Women who scored ≥10 on the EPDS were referred to the nearby district hospital for adequate follow-up [52]. The interviewers also aimed to recognize and counter any feelings of distress among the identified abused women by describing how their participation in the study might contribute to better understanding of IPV and its influence on women’s health and well-being.

### Human participation protection

Ethical approval was received for scientific and ethical integrity from the National research ethics committee of Bangladesh Medical Research Council (BMRC/NREC/2013-2016/305) and Griffith University Human Research Ethics Committee (CCJ/41/14/HREC) before conducting the study. The study was also conducted following the WHO guidelines on ethical issues for violence research [53]. Interviewers read out the informed consent form in front of the respondents at the beginning of the study. Each respondent was informed about the objectives of the study and the process of maintaining confidentiality and anonymity of their personal information. In consideration of the sensitive nature and cultural context of the study, verbal informed consent from respondents was obtained to ensure complete anonymity for respondents and to avoid any legal consequences.

### Outcome variable

The main outcome of the present study was postpartum depression assessed by using the Bangla version of the Edinburgh Postpartum Depression Scale (EPDS) [54]. The EPDS comprises 10 items with four response categories scored from 0 to 3 and was used to detect symptoms of depression in the first six months postpartum. Postpartum mothers were asked whether they experienced the following feelings in the past seven days of the interview: ‘I have been able to laugh and see the funny side of things’, ‘I have looked forward with enjoyment to things’, ‘I have blamed myself unnecessarily when things went wrong’, ‘I have been anxious or worried for no good reason’, ‘I have felt scared or panicky for no very good reason’, ‘things have been getting on top of me’, ‘I have been so unhappy that I have had difficulty sleeping’, ‘I have felt sad or miserable’, ‘I have been so unhappy that I have been crying’, and ‘the thought of harming myself has occurred to me’. A higher score refers to a higher depressed mood. The Bangla version of the EPDS has shown a sensitivity of 89% and a specificity of 87% at the optimum cut-off score of 10 [55]. Following this, the cut-off score was set at 10 points or more to define clinically significant symptoms of postpartum depression in the present study. Women were then classified as non-depressed (score <10 = 0) and depressed (score ≥10 = 1). The internal consistency for this scale was very good (Cronbach’s α = .90).

Each participant was asked if she had experienced any of these symptoms during pregnancy or before, referred to herein as previous depressive symptoms (categorized as: no = 0 and yes = 1).

### Exposure to intimate partner violence

Women’s experience of IPV before, during and after pregnancy was the main research interest, and for the purpose of the study ‘intimate partner’ refers to the respondent’s current spouse. The study collected information on IPV experienced by women before, during and after pregnancy using the domestic violence module from the WHO’s Demographic Health Survey Questionnaire. The domestic violence module used in the WHO study was validated for use in Bangladesh and some other countries [56]. A positive answer to any one of the following seven behaviours constitute physical IPV in this study: (1) pushing, shaking, or throwing something at her; (2) slapping; (3) twisting her arm or pulling her hair; (4) punching or hitting with a fist or something harmful; (5) kicking or dragging or physically assaulting her; (6) choking or burning; or (7) threatening or attacking with a knife, gun or any other weapon.

A woman was coded as having experienced sexual violence by an intimate partner if she reported having been physically forced to have sexual intercourse; having intercourse out of fear, or being forced to perform other sexual acts that she found degrading or humiliating. Psychological IPV was measured by at least one affirmative response to questions asking whether or not the respondent’s husband had insulted her or made her feel bad about herself; humiliated her in front of others; threatened to hurt her or someone close to her; isolated her from friends and family; denied her access to money or other basic resources; or threatened to divorce her. The Cronbach’s alphas for physical, sexual and psychological IPV scale in this study were 0.78, 0.47 and 0.75, respectively.

Each participant was asked if she had experienced any of these indicators of IPV during the first six months after birth of her last baby, during her pregnancy, and during the 12 months prior to pregnancy, referred to herein as ‘IPV after pregnancy’, ‘IPV during pregnancy’ and ‘IPV before pregnancy’, respectively. Physical, sexual and psychological IPV before, during and after pregnancy were coded as no (= 0) and yes (= 1).

### Control variables

Several socio-demographic variables that have been theoretically and empirically associated with IPV and PPD [49, 57–62] were included in this study to control for their influence on the primary relationships of interest. The maternal age during the last pregnancy was categorized into three groups, roughly representative of adolescence, young adulthood and adulthood (14–18 years = 0, 19–24 years = 1, or 25 years and over = 2). The maternal educational level was classified with regard to the formal education system of Bangladesh: no education (0 years = 0), primary (1–5 years = 1), and secondary and higher (6 years or more = 2). Family monthly income was classified according to the national average (8500 BDT ~ 109 USD) as ≤ 8500 BDT (= 0) versus > 8500 BDT (= 1).

Obstetric and reproductive characteristics such as pregnancy intention (unintended = 0, intended = 1), parity (primiparous = 0, multiparous = 1), number of children under five years of age (1 = 0, 2 = 1), complications during childbirth (no = 0, yes = 1), mode of birth (caesarean = 0, vaginal = 1), a husband’s preference for a son (no = 0, yes = 1) and timing of breastfeeding initiation (immediately = 0, late = 1) were taken into consideration. Regarding psycho-socio-cultural characteristics, to ascertain the relationship with their mother-in-law, women were asked to evaluate their relationship with their mother-in-law providing a score from 1 to 9, where higher score indicates a more positive relationship (bad, 1–3 points = 0, mild (4–6 points) = 1, good (7–9 points) = 2).

#### Social support

We control for social support given its protective impacts on mental health in general and PPD in particular [44, 50, 63]. Chan et al. [64] adopted a 10-item social support scale from the Family Needs Screener (a short version of personal and relationship profile prepared by Straus and associates [65]). Women were asked to respond using four response categories (1 = strongly disagree, 2 = disagree, 3 = agree, and 4 = strongly agree) to the 10 statements (‘only have a limited number of friends or family members to help with baby/children’, ‘feel very lonely’, ‘someone makes me feel confident’, ‘someone I can talk to frankly’, ‘someone I can talk to regarding my personal problems’, ‘I have someone to borrow money from in a financial difficulty’, ‘have someone to look after my children’, ‘have someone who helps me around the house’, ‘have someone I can count on in times of need’, and ‘don’t have enough money for my daily needs’). Women with total scores in the bottom third were classified as having low social support (= 0), those in the middle third as evidencing medium social support (= 1), and those in the top third as showing high social support (= 2). The internal consistency for this scale was very good (Cronbach’s α = .90).

#### Perceived stress

Stress can increase the odds of PPD [66, 67], so models control for its influence as well. Respondent stress levels were measured with the Perceived Stress Scale (PSS). The PSS is a 10-item self-report questionnaire generally used to measure the degree to which situations in one’s life are appraised as stressful [68]. Women were asked to respond using five response categories (0 = never, 1 = almost never, 2 = disagree, 3 = sometimes, 4 = fairly often and 5 = often) to the 10 questions (in the last month, how often have you ‘been upset because of something that happened unexpectedly?’, ‘felt that you were unable to control the important things in your life?’, ‘felt nervous and Stressed?’, ‘felt confident about your ability to handle your personal problems?’, ‘felt that things were going your way?’, ‘found that you could not cope with all the things that you had to do?’, ‘been able to control irritations in your life?’, ‘felt that you were on top of things?’, ‘been angered because of things that were outside your control?’, and ‘felt difficulties were piling up so high that you could not overcome them?’). Higher scores represent increased stress. After reverse scoring for some items, a total score ranges from 0–40. The Bangla version of PSS was validated in Bangladesh [69]. The PSS is not a diagnostic instrument and there are no predetermined cut-points [70]. For our analysis, the cut-off score was set at 20 points to classify women as low stressed (score <20 = 0) and high stressed (score ≥20 = 1) in accordance with a study from Pakistan [71]. In the present study, the Cronbach’s αfor this scale was .93.

#### Self-esteem

High self-esteem can also protect against PPD [72]. To control for this influence, women’s self-esteem was measured with the Rosenberg self-esteem scale (RSES). The RSES is a 10-item scale that assesses self-esteem by measuring both positive and negative feelings about the self [73]. All items are answered using a 4-point Likert scale format ranging from strongly agree to strongly disagree (‘on the whole, I am satisfied with myself’, ‘at times, I think I am no good at all’, ‘I feel that I have a number of good qualities’, ‘I am able to do things as well as most other people’, ‘I feel I do not have much to be proud of’, ‘I certainly feel useless at times’, ‘I feel that I am a person of worth, at least the equal of others’, ‘I wish I could have more respect for myself’, ‘all in all, I am inclined to feel that I am a failure’, and ‘I take a positive attitude toward myself’). After reverse scoring for some items, a total score ranges from 0–30. Higher scores refer to higher self-esteem. The Bangla version of PSS was validated in Bangladesh [69]. No predetermined cut-points for low self-esteem exist [74]. However, the cut-off score was set for this analysis at 15 points to classify women as low self-esteemed (score <15 = 0) and high self-esteemed (score ≥15 = 1) following the guidelines of some organizations [75, 76]. The internal consistency for this scale was very good in the present study (Cronbach’s α = .84).

#### Husband’s controlling behaviors

Women with more controlling husbands are at risk for both IPV and PPD, an influence we control with a scale comprised of the following items: (a) husband tries to keep her from seeing friends; (b) tries to restrict contact with her family of birth; (c) insists on knowing where she is at all times; (d) does not trust her with any money; (e) gets angry if she speaks to another man; (f) is often suspicious that she is unfaithful; and (g) expects her to ask permission before seeking health care for herself. The responses to these variables are dichotomous (0 = no and 1 = yes). Based on the total scores, women with scores in the bottom third percentile were classified as having husbands who were less controlling (= 0), those with scores in the middle third percentile as having moderately controlling husbands (= 1), and those in the top third as having husbands who were highly controlling (= 2). The controlling behaviors scale was validated for use in Bangladesh and some other countries [77]. The Cronbach’s alpha for this scale in the present study was .72.

#### Women’s decision-making autonomy

Women who experience a higher degree of autonomy are less likely to report IPV or PPD [78]. We control for this with a scale that reflects the number of household decisions a woman made alone or jointly with her husband about: (1) spending her income; (2) obtaining health care for herself; (3) major household purchases; (4) purchases for daily household needs; (5) visit to family or relatives; and (6) obtaining child health care [79]. The response options were: (a) respondent alone, (b) respondent and husband, (c) respondent and someone else, (d) husband alone, (e) someone else. Each question was assigned a value of 1 if the response was (a), (b), or (c), and 0 for (d), or (e). The scores were summed together resulting in a score from 0 to 6 (Cronbach’s alpha = .87). Based on the total scores, women were classified into one of three groups. This with scores in the bottom third percentile were categorized as having low decision-making autonomy (= 0), women with scores in the middle third percentile were classified as having moderate decision-making autonomy (= 1), and those with scores in the top third percentile were categorised with high decision-making autonomy (= 2).

### Statistical analysis

SPSS version 22.0 for Windows (SPSS Inc., Chicago, IL, USA) was used for coding and analyzing the data. We calculated descriptive statistics for mother’s postpartum depressive symptoms, and exposure to IPV as well as all of our control variables. Our analysis proceeded in two stages. First, we conducted a series of cross-tabulations to assess the bivariate relationships between PPD and the relevant covariates and controls and report chi-squares as our measure of significance. A two-tail p-value of <.05 was set to refer the level of statistical significance for all analyses. We then proceeded to a series of multivariate models that allow us to assess the strength of any association between IPV and PPD, controlling for a range of other known influences. These models employed multivariate logistic regression to estimate adjusted odds ratios (AOR) and 95% confidence intervals to compare the strength of the associations between each of the covariates and PPD. To assess the influence of IPV after childbirths on the odds of PPD, we ran four adjusted multivariate logistic regression models—one for each type of IPV after childbirth to assess the separate effects of different forms of IPV on PPD, and one full model for all types of IPV together with the control variables to examine the effects of each type of IPV, controlling for the other. Each model also included the control variables described above. A second set of adjusted multivariate logistic regression models examined the influence of IPV before and during pregnancy on PPD outcomes. Here we ran one model for all types of IPV victimization before pregnancy and another for all types of IPV victimization during pregnancy to examine whether past IPV victimization is associated with experiencing PPD. The multicollinearity of the variables was tested by auditing the variance inflation factors (VIFs), but there was no evidence that this was a problem (VIF’s<2.5).

## Results

### Profile of the respondents

Table 1 reports the characteristics of the women in our sample and documents the distribution of PPD among women by these characteristics. Approximately quarter of the women (24.9%) were 14–18 years old, just less than half of the women (43.9%) were 19–24 years old and one-third or the women (31.2%) were 25 years or over during their pregnancy. Nearly 8.2% of women had no formal education and 67.4% had either secondary or higher education. About 38.3% women had an average monthly income less than or equal to the national average. About 41.1% of women had one child and nearly one-fifth of women (22.1%) had two children less than five years of age. One fourth of the last pregnancies was unintended. More women (69.2%) in this sample gave birth via vaginal delivery than by caesarean delivery (30.8%). About 16.2% of women had complications during childbirth and just over half (56.8%) of the women could initiate breastfeeding within 24h. Furthermore, approximately, 43.2% of women’s husband had a preference for a son.

**Table 1:** Sociodemographic and other characteristics of women, and bivariate association of these characteristics with postpartum depression among women of Bangladesh (*N* = 426).

Characteristics	*n* (%)	Postpartum depression	
		%	*p*-value
**Socio-demographic characteristics**			
Age during pregnancy			
14–18	106 (24.9)	26.0	.35
19–24	187 (43.9)	39.3	
≥ 25	133 (31.2)	34.7	
Maternal education			
No formal education	35 (8.2)	62.9	<.001
Primary	104 (24.4)	48.1	
Secondary and higher	287 (67.4)	27.2	
Family monthly income, BDT			
≤8500	163 (38.3)	52.1	<.001
>8500	263 (61.7)	24.7	
**Obstetric and reproductive characteristics**			
Parity			
Primiparous	175 (41.1)	30.3	.08
Multiparous	251 (58.9)	38.6	
Number of under-five children			
1	332 (77.9)	33.1	.09
2	94 (22.1)	42.6	
Pregnancy intention^¥^			
Unintended	107 (25.1)	43.9	.03
Intended	319 (74.9)	32.3	
Complications during childbirth			
No	357 (83.8)	33.9	.20
Yes	69 (16.2)	42.0	
Mode of childbirth			
Caesarean section	131 (30.8)	25.2	.004
Vaginal	295 (69.2)	39.7	
Husband’s son preference			
No	242 (56.8)	46.7	.002
Yes	184 (43.2)	53.3	
Breastfeeding initiation			
Immediately	249 (58.5)	31.7	.07
Late	177 (41.5)	40.1	
**Psycho-socio-cultural characteristics**			
Women decision-making autonomy			
Low	110 (25.8)	62.7	<.001
Moderate	175 (41.1)	41.1	
High	141 (33.1)	6.4	
Relationship with mother-in-law			
Bad	126 (29.6)	54.8	<.001
Mild	188 (44.1)	33.5	
Good	112 (26.3)	16.1	
Husband’s controlling behaviors			
Less	142 (33.3)	8.5	<.001
Moderate	166 (39.0)	41.6	
High	118 (27.7)	58.5	
Previous depressive symptoms			
No	312 (73.2)	22.4	<.001
Yes	114 (26.8)	70.2	
Perceived stress			
Low	183 (43.0)	11.5	<.001
High	243 (57.0)	53.1	
Social support			
Low	128 (30.0)	68.0	<.001
Medium	158 (37.1)	35.4	
High	140 (32.9)	5.0	
Self-esteem			
Low	249 (58.5)	55.0	<.001
High	177 (41.5)	7.3	
IPV before pregnancy			
Any physical			
No	201 (47.2)	7.0	<.001
Yes	225 (52.8)	60.4	
Any sexual			
No	336 (78.9)	33.6	.19
Yes	90 (21.1)	41.1	
Any psychological			
No	139 (32.6)	5.0	<.001
Yes	287 (67.4)	49.8	
All types			
No	365 (85.7)	31.5	<.001
Yes	61 (14.3)	57.4	
IPV during pregnancy			
Any physical			
No	276 (64.8)	18.1	<.001
Yes	150 (35.2)	66.7	
Any sexual			
No	347 (81.5)	28.8	<.001
Yes	79 (18.5)	63.3	
Any psychological			
No	149 (35.0)	2.7	<.001
Yes	277 (65.0)	52.7	
All types			
No	378 (88.7)	29.6	<.001
Yes	48 (11.3)	79.2	
IPV after childbirth			
Any physical			
No	289 (67.8)	17.0	<.001
Yes	137 (32.2)	73.7	
Any sexual			
No	360 (84.5)	31.4	<.001
Yes	66 (15.5)	56.1	
Any psychological			
No	167 (39.2)	7.8	<.001
Yes	259 (60.8)	52.9	
All types			
No	387 (90.8)	31.5	<.001
Yes	39 (9.2)	71.8	
	**Total**	**35.2**	

^¥^ Intended: live birth wanted at the time of conception or unintended: live birth wanted later or not at all.

Approximately, a quarter of women (25.8%) had low decision-making autonomy and one-third of the women (29.6%) reported relationship difficulties with their mother-in-law. About, one in three (27.7%) women’s husband had high controlling behaviors and 26.8% of women reported a prior history of depressive symptoms. Approximately 57% of women had high perceived stress, 58.5% of women had low self-esteem and nearly one-third of the women (30%) had low social support.

The prevalence of physical IPV during the 12 months before the pregnancy was 52.8%, while the comparative figure for during pregnancy and after childbirth was 35.2% and 32.2% respectively. The prevalence of psychological IPV before pregnancy was highest (67.4%) compared with that during pregnancy (65%) and after childbirth (60.8%). The rate of sexual IPV after childbirth was lower (15.5%) than that of before (21.1%) and during (18.5%) the pregnancy.

**Fig 1:**
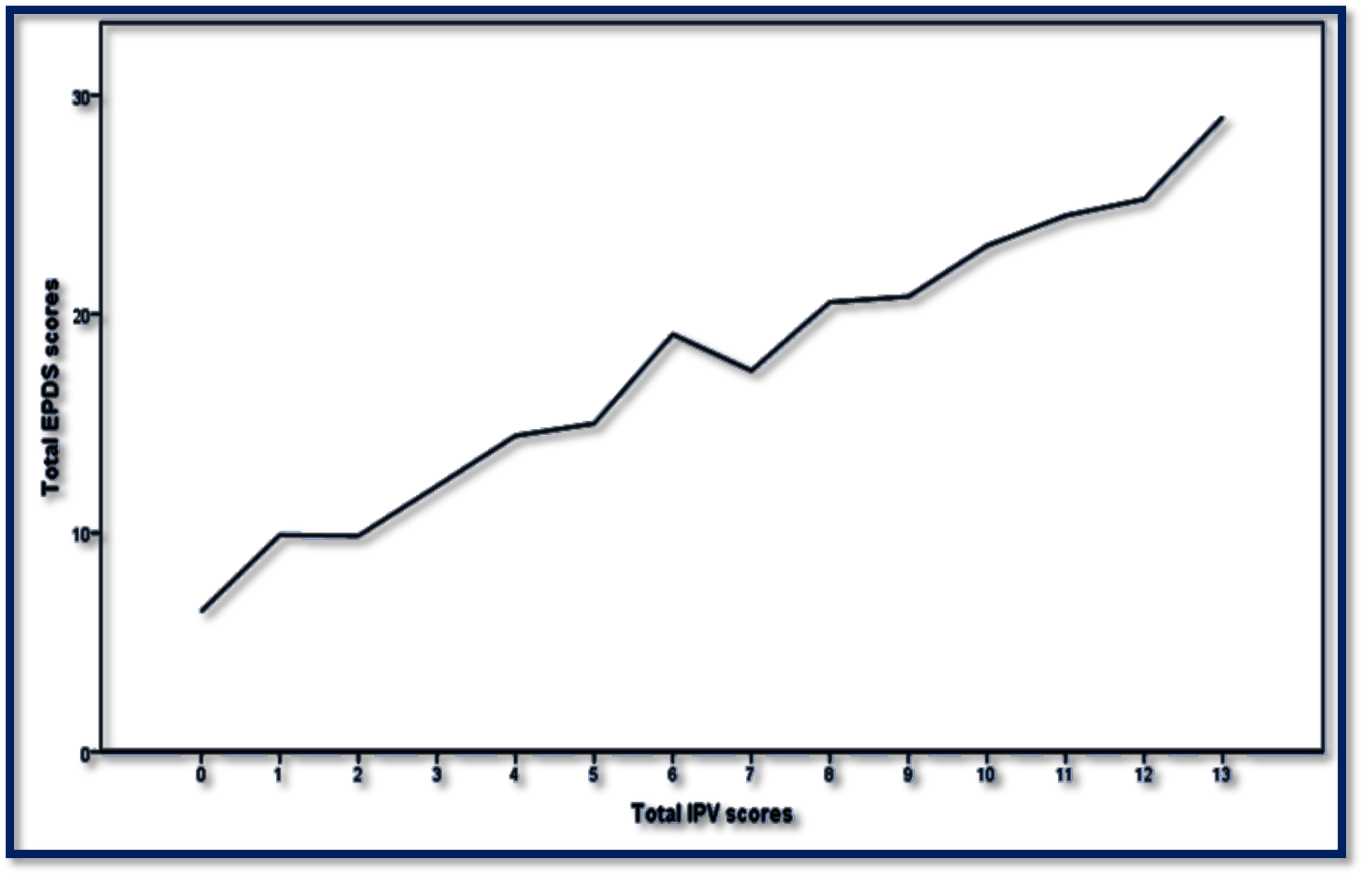
Association between total EPDS scores and total IPV scores after childbirth among new mothers in Bangladesh.

**Fig 2:**
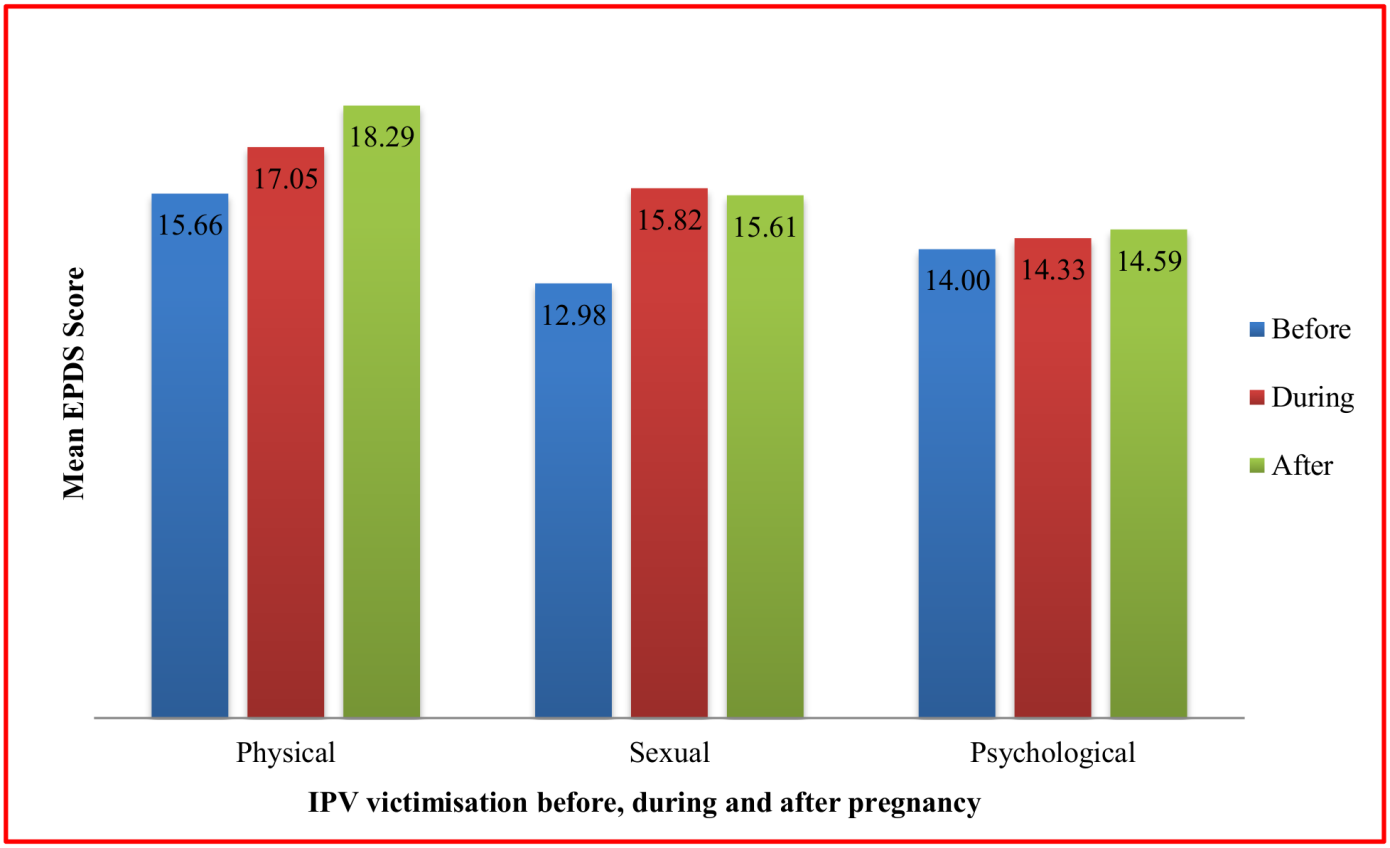
Mean EPDS scores among depressed mothers depending on the timing of IPV occurrence.

Out of 426 participants, 150 had total EPDS scores of 10 or above, indicating the prevalence of PPD is 35.2%. Fig 1 shows that there was a linear association between total EPDS scores and total IPV scores. Fig 2 shows mean EPDS score among depressed women based on the timing of IPV occurrence. On average, the mean EPDS scores were the highest for women who had experienced physical IPV, followed by sexual and psychological IPV victimization. More specifically, the mean EPDS scores were the highest (18.29) for women with physical IPV victimization after childbirth, followed by physical IPV victimization during (17.05) and before pregnancy (15.66).

### IPV and postpartum depression: Bivariate associations

We begin by describing the bivariate relationships between the forms and timing of IPV and PPD outcomes among new mothers. As expected, physical, sexual, and psychological IPV both during pregnancy and after childbirth were significantly associated with PPD among the women in this sample. When we examine IPV experiences prior to pregnancy, physical and psychological IPV increase the odds of PPD, but that is not the case for sexual IPV. Overall, 73.7% of women who experienced physical IPV, 56.1% of those experiencing sexual IPV and 52.9% of women experiencing psychological IPV after childbirth also reported PPD. Similarly, during pregnancy, approximately two-thirds of women who experienced physical IPV (66.7%) and sexual IPV (63.3%) and nearly half of the women (52.7%) who experienced psychological IPV also reported PPD. Prior to pregnancy, the corresponding figures were 60.4%, 41.1% and 49.4%, respectively who reported PPD. Moreover, approximately 57.4%, 79.2% and 71.8% of women who experienced all types of IPV before, during and after pregnancy respectively also reported PPD. Bivariate results (see Table 1) also reinforce the importance of controlling for numerous factors that influence women’s likelihood of PPD. We turn now to multivariate models that take these relationships into account.

### IPV and postpartum depression: Multivariate analysis

Results reported in Table 2 illustrate outcomes of multivariate models examining the effects of IPV after childbirth on experiencing PPD controlling for the range of socio-demographic, obstetric and reproductive, and psycho-socio-cultural influences included in the survey. We examine each type of IPV independently (Table 2, columns 2–4) and then jointly (Table 2, column 5). The joint model allows us to assess whether the influence of any particular form of IPV holds controlling for other forms of IPV. This is important given overlap across forms of IPV. Model 1–3 demonstrates that only physical IPV after childbirth significantly influences PPD. With the introduction of all forms of IPV with control variables in the full model (Table 2, column 5), the odds ratios of physical IPV (AOR: 4.01, 95% CI [2.07, 7.76]) remains significant. Women who experience physical IPV during the first six months following childbirth are 4.01 times more likely to experience PPD than women who do not experience such violence. In contrast, experiencing sexual and psychological IPV after childbirth does not significantly influence the odds of PPD in the full model.

**Table 2:** Logistic Regression Odds ratios for the association between IPV victimization after childbirth and the postpartum depression (*N* = 426).

Independent variable	Postpartum Depression, AOR (95% CI)			
	Model 1	Model 2	Model 3	Model 4 (Full model)
**Any physical IPV**				
No	1.00	-	-	1.00
Yes	4.13 (2.15–7.93)*	-	-	4.01 (2.07–7.76)*
**Any sexual IPV**				
No	-	1.00	-	1.00
Yes	-	1.25 (0.64–2.45)	-	1.00 (0.49–2.03)
**Any psychological IPV**				
No	-	-	1.00	1.00
Yes	-	-	1.92 (0.78–4.71)	1.61 (0.62–4.17)
**Previous depressive symptoms**				
Non-depressive symptoms	1.00	1.00	1.00	1.00
Depressive symptoms	2.61 (1.40–4.85)**	2.80 (1.55–5.06)*	2.78 (1.54–5.03)*	2.58 (1.38–4.80)**
**Perceived stress**				
Low	1.00	1.00	1.00	1.00
High	2.10 (1.00–4.43)***	2.69 (1.31–5.51)**	2.56 (1.24–5.26)**	2.02 (1.55–4.30)***
**Husband’s controlling behaviors**				
Less	1.00	1.00	1.00	1.00
Moderate	0.91 (0.31–2.64)	0.95 (0.34–2.65)	0.84 (0.29–2.41)	0.80 (0.26–2.45)
High	0.94 (0.30–2.93)	0.99 (0.34–2.93)	0.86 (0.28–2.65)	0.83 (0.25–2.70)
**Social support**				
Low	1.00	1.00	1.00	1.00
Medium	0.49 (0.26–0.94)***	0.44 (0.24–0.80)**	0.44 (0.24–0.81)**	0.50 (0.26–0.95)***
High	0.19 (0.05–0.64)**	0.15 (0.04–0.50)**	0.16 (0.05–0.58)**	0.20 (0.06–0.73)**
**Self-esteem**				
Low	1.00	1.00	1.00	1.00
High	0.72 (0.26–1.97)	0.60 (0.23–1.61)	0.65 (0.24–1.75)	0.73 (0.25–2.07)
**Maternal age during pregnancy**				
14–18	1.00	1.00	1.00	1.00
19–24	0.66 (0.30–1.46)	0.65 (0.30–1.41)	0.63 (0.29–1.37)	0.66 (0.29–1.48)
≥25	1.06 (0.41–2.78)	1.16 (0.46–2.97)	1.11 (0.44–2.84)	1.06 (0.40–2.81)
**Maternal education**				
No formal education	1.00	1.00	1.00	1.00
Primary	0.26 (0.09–0.76)**	0.35 (0.13–1.00)***	0.38 (0.13–1.09)	0.27 (0.09–0.81)***
Secondary and higher	0.33 (0.12–0.92)***	0.41 (0.15–1.10)	0.44 (0.16–1.20)	0.35 (0.12–0.99)***
**Family monthly income, BDT**				
≤8500	1.00	1.00	1.00	1.00
>8500	1.09 (0.58–2.04)	1.00 (0.55–1.84)	1.02 (0.56–1.89)	1.09 (0.57–2.07)
**Parity**				
Primiparous	1.00	1.00	1.00	1.00
Multiparous	1.42 (0.61–3.32)	1.58 (0.70–3.59)	1.63 (0.72–3.71)	1.45 (0.62–3.40)
**Pregnancy intention**^¥^				
Unintended	1.00	1.00	1.00	1.00
Intended	1.49 (0.77–2.86) . (0.73–2.03)	1.39 (0.74–2.62)	1.37 (0.72–2.59)	1.47 (0.76–2.83)
**Number of children aged under five years**				
1	1.00	1.00	1.00	1.00
2	0.95 (0.44–2.06)	0.75 (0.36–1.56)	0.75 (0.36–1.57)	0.95 (0.44–2.06)
**Husband’s preference for a son**				
No	1.00	1.00	1.00	1.00
Yes	1.98 (1.11–3.54)***	1.82 (1.05–3.17)***	1.76 (1.01–3.07)***	1.93 (1.07–3.46)***
**Relationship with mother-in-law**				
Bad	1.00	1.00	1.00	1.00
Medium	1.05 (0.55–2.02)	1.01 (0.54–1.87)	1.04 (0.56–1.95)	1.09 (0.57–2.08)
Good	1.88 (0.71–4.95)	1.69 (0.67–4.21)	1.93 (0.75–4.96)	2.06 (0.76–5.58)
**Mode of childbirth**				
Caesarean section	1.00	1.00	1.00	1.00
Vaginal	1.73 (0.77–3.87)	1.50 (0.70–3.22)	1.57 (0.73–3.38)	1.77 (0.79–3.97)
**Complications during childbirth**				
No	1.00	1.00	1.00	1.00
Yes	3.58 (1.53–8.41)**	3.42 (1.48–7.89)**	3.29 (1.45–7.51)**	3.53 (1.51–8.26)**
**Breastfeeding initiation**				
Immediately	1.00	1.00	1.00	1.00
Late	1.31 (0.69–2.50)	1.39 (0.74–2.61)	1.44 (0.76–2.70)	1.31 (0.68–2.55)
**Women’s decision-making autonomy**				
Low	1.00	1.00	1.00	1.00
Moderate	0.82 (0.44–1.54)	0.71 (0.39–1.29)	0.72 (0.39–1.31)	0.83 (0.44–1.57)
High	0.30 (0.10–0.89)***	0.20 (0.07–0.57)**	0.22 (0.07–0.63)**	0.32 (0.10–0.99)***

Note: AOR = Adjusted Odds Ratio; CI = Confidence Interval; IPV = Intimate partner violence.

^¥^ Intended: live birth wanted at the time of conception or unintended: live birth wanted later or not at all.

Here *p<0.001; **p<0.01; ***p<0.05

Model 1: influence of physical IPV after childbirth adjusted for maternal age during pregnancy, maternal education, income, parity, number of under-five children, pregnancy intention, husband’s preference for a son, relationship with mother-in-law, mode of childbirth, complications during childbirth, breastfeeding initiation, women’s decision-making autonomy, social support, self-esteem, previous depressive symptoms, perceived stress and husband’s controlling behaviours.

Model 2: influence of sexual IPV after childbirth adjusted for the above variables.

Model 3: influence of psychological IPV after childbirth adjusted for the above variables.

Model 4: influence of all types of IPV after childbirth adjusted for the above variables.

As is evidenced in Table 2, controls generally operate as expected, most notably, stress and lack of social support increase women’s likelihood of PPD. These are particularly important given their known associations with IPV [80–83]. We found no significant association in the multivariate analysis between experiencing PPD and maternal age during pregnancy, family monthly income, parity, pregnancy intention, the number of children less than five years of age, relationship with mother-in-law, mode of childbirth, the timing of breastfeeding initiation, self-esteem and husband’s controlling behaviors.

Similar to the influence of IPV victimization after childbirth, results reported in Table 3 (Model I) show that only physical IPV victimization before pregnancy significantly increases the odds of experiencing PPD after adjusting for all other forms of IPV along with socio-demographic, obstetric and reproductive, and psycho-socio-cultural factors. In fact, women who experience physical IPV before pregnancy were 3.53 times (95% CI [1.40, 8.88]) more likely to experience PPD than women who had not experienced such violence before pregnancy.

**Table 3:** Logistic Regression Odds ratios for the association between IPV victimization before and during pregnancy and PPD (*N* = 426).

	Independent variables	Postpartum Depression, AOR (95% CI)
**Model I**	**IPV Before Pregnancy**	
	Any physical	
	No	1.00
	Yes	3.53 (1.40–8.88)**
	Any sexual	
	No	1.00
	Yes	0.80 (0.41–1.59)
	Any psychological	
	No	1.00
	Yes	1.99 (0.56–7.14)
**Model II**	**IPV during pregnancy**	
	Any physical	
	No	1.00
	Yes	1.79 (1.25–3.43)***
	Any sexual	
	No	1.00
	Yes	2.25 (1.14–4.45)*
	Any psychological	
	No	1.00
	Yes	6.92 (1.71–28.04)**

Model I: influence of all types of IPV before pregnancy adjusted for all other socio-demographic, obstetric and reproductive, and psycho-socio-cultural factors used in this study.

Model II: influence of all types of IPV during pregnancy adjusted for all other socio-demographic, obstetric and reproductive, and psycho-socio-cultural factors.

Here *p<0.001; **p<0.01; ***p<0.05

During pregnancy, the picture looks notably different. At this stage, physical, sexual and psychological IPV victimization were all found to be independent risk factors for experiencing PPD after adjusting for other control variables (see Table 3, Model II). Overall, women who experience physical IPV and sexual IPV during pregnancy were respectively 1.79 times (95% CI [1.25, 3.43]) and 2.25 times (95% CI [1.14, 4.45]) more likely to experience PPD than women who had not experienced such violence during pregnancy. Even more concerning is the particularly strong influence of psychological IPV during pregnancy on PPD outcomes. The model indicates that the odds of PPD are 6.92 times (95% CI [1.71, 28.04]) higher for women who experience psychological abuse during pregnancy compared to those who do not report such abuse.

## Discussion

In this study, we examined the influence of physical, sexual and psychological IPV victimization before, during and after pregnancy on postpartum depressive symptoms in a population-based sample of women of Bangladesh using a retrospective, cross-sectional design. Although a range of studies have explored associations between exposure to IPV during one period and PPD [43], this study contributes to the empirical literature by demonstrating individual effects of physical, sexual and psychological IPV experienced during three different periods (before, during and after pregnancy), on PPD. IPV is clearly a significant problem in Bangladesh [27], and findings from the current study certainly indicate that PPD is a similarly significant problem. Just above one-third of new mothers in this sample reported symptoms consistent with postpartum depression during the first six months after childbirth. Our analyses clearly suggest that these two problems have significant overlap, which has implications for prevention and intervention with both IPV and PPD.

The prevalence of PPD was 35.2% in this study, which is higher than the 10–20% prevalence demonstrated in some systematic reviews [8, 84, 85]. One literature review [62] showed that the prevalence of PPD ranged anywhere from 3.5% to as high as 63.3% across Asian countries depending on the definitions used. However, there appears to be some consistency in prevalence rates in a range of studies conducted in various developing countries. For example, the prevalence of PPD was 38.3% in Pakistan [86], 33.2% in Turkey [87], 33% in Vietnam [88], 33% in Zimbabwe [89] and 27.9% in Brazil [90]. Using the EPDS tool, limited PPD studies in Bangladesh reported the prevalence of PPD to be 22% at 6–8 weeks postpartum [52] and 32% at 6–8 months postpartum [91]. Our PPD prevalence estimate is nearly in accordance with these findings.

Findings from this community-based study of women of Bangladesh revealed that the prevalence of physical IPV during the 12 months before the pregnancy was 52.8%, while the comparative figures for during pregnancy and after childbirth were 35.2% and 32.2% respectively. The prevalence of sexual IPV before, during and after pregnancy was found to be 21.1%, 18.5% and 15.5% respectively. The corresponding figures for psychological IPV were 67.4%, 65% and 60.8% respectively. These prevalence rates of IPV are in line with other studies from Bangladesh which have reported the past year or current prevalence of physical IPV to be between 16–52% [91–94], sexual IPV between 11–65% [77, 91, 95, 96] and psychological IPV between 24–84% [91, 92]. The reported rate of physical IPV during pregnancy (35.2%) is higher than reported in a multi-country study as of between 2% and 13.5% [34]. Such findings confirm that IPV in Bangladesh is alarmingly commonplace.

Our primary interest in this study was in assessing the link between IPV exposure and PPD. Previous studies have suggested that women who experienced physical or sexual IPV before, during or after pregnancy had higher levels of postpartum depressive symptoms than non-victims [40, 45, 58, 97–99]. In this study, we find a particularly enduring association between physical IPV and PPD. Among the new mothers in our study, exposure to physical IPV before, during and after pregnancy increases the odds of postpartum depressive symptoms, even after controlling for key variables, including other forms of IPV. This comports with a range of studies linking physical abuse to PPD [40, 45, 58]. However, as our findings reinforce, other IPV experiences can also increase the likelihood of PPD, especially when they occur during pregnancy. Although sexual and psychological IPV before and after pregnancy do not increase the odds of PPD among the mothers in our sample, exposure to any form of IPV (physical, psychological and/or sexual) during pregnancy significantly increased the odds of postpartum depression. We are not the first to find that the timing of IPV around pregnancy is relevant to the likelihood of PPD outcomes. A number of studies document that psychological abuse during pregnancy is particularly consequential for PPD relative to its influence before pregnancy [40, 42, 98, 100–104]. However, similar to our findings with women of Bangladesh, Martin et al. (2006) report that psychological IPV during the year prior to pregnancy did not increase PPD symptoms among a sample of US women.

The importance of both the type and timing of IPV for PPD outcomes is notable. Clearly, physical IPV around the time of pregnancy introduces a variety of risks to the health and safety of the mother and it is not surprising that it increases PPD symptoms regardless of when in the pregnancy timeline a woman experiences it. The distal and contemporaneous effect of physical IPV on the mental health of new mothers is consistent with our expectations. More surprising is the finding that the effect of psychological and sexual abuse on PPD is limited to exposure during pregnancy. We find this regardless of whether we control for the influence of physical IPV, so this does not simply reflect the overlapping nature of the three forms of abuse. Certainly, this does not mean sexual and psychological abuse outside the pregnancy period are not problematic, we know from a large body of literature that these forms of abuse have multiple deleterious effects on the lives of women [43, 60, 105, 106]. But, where PPD is concerned, they are particularly consequential during pregnancy. It may be that women are especially vulnerable to the effects of all types of IPV in this stage because of their heightened concern for the health and safety of their developing child. Indeed, literature links women’s feelings of inadequacy around parenting to PPD [94, 107–110] and psychological and sexual abuse during pregnancy may inflate such concerns. Additionally, prior to and after pregnancy, it may be easier for a mother to minimize the potential influence of spousal psychological and sexual abuse on her child, compared to physical abuse, which she might worry, would also extend to her child. Qualitative research exploring these links would help tease out some of these potential dynamics and would likely introduce other explanations.

Our cross-sectional design does not allow us to examine time-ordered associations between abuse and some of the mechanisms that may explain its links to subsequent PPD. However, we suspect that some of the mechanisms we control for here might be implicated in this process. We know for instance that women with reduced social support are more likely to report symptoms of PPD [40, 50, 58, 63, 66, 90, 111]. It may be that women, who experience any form of IPV during pregnancy feel ashamed or worry thinking that others will judge them for not adequately protecting their developing child and so, cut themselves off from others, aggravating the likelihood of later PPD. These experiences may also heighten stress levels among women, which contribute to PPD [50, 66, 67, 112]. Panel data would be necessary to assess these time ordered processes.

### Limitations and strengths of the study

Martin et al. (2006) highlighted that more research utilizing a longitudinal approach is warranted to examine how IPV victimization not only before and during pregnancy but also after childbirth may affect women’s mental health. Though our study is not longitudinal, it includes retrospective and time ordered measures of abuse, thus offering some insight into the longitudinal links between IPV and PPD. To our knowledge, this is the first study from South Asia, particularly from Bangladesh that specifically examined the association of IPV with postpartum depressive symptoms as a function of the type and timing of IPV occurrence. The strength of the study is the large sample size and the introduction of a large number of different socio-demographic, obstetric and reproductive, and psycho-socio-cultural factors. In addition, it is one of the few studies that included various types of IPV experienced at different time periods to explore the likelihood of experiencing PPD. Most importantly, by using internationally recognized scales and questionnaire for this study, the findings are internationally comparable. It is also important to acknowledge that the potential recall bias of the particular cohort of women in this study is greatly reduced because the mothers were surveyed within six months of the birth. Nevertheless, our findings should be interpreted while acknowledging several limitations to the study. Specifically, due to cross-sectional nature of this study, it is not possible to determine the exact temporal relationship between exposure to IPV and PPD. Neither, did we focus on the frequency of IPV over time. In addition, although we collected information on violence and depression before and during pregnancy and in the postpartum period, because the data are cross-sectional it is possible that women’s current depression and violence experiences influence their recollection of prior violence and depressive symptoms. However, regardless of the limitations, we do see changes in these experiences across the three periods, which would indicate that most women are able to disentangle their current state from previous ones and respond accordingly. It is also relevant that the data were collected from one district only, therefore, the findings may not be considered to be representative of the general population. Although a substantial number of predictors were adjusted in this study, some of the important variables were not controlled for, such as the health of the baby and difficult infant temperament. Finally, due to budget and time constraints, we were only able to collect data on exposure to IPV before, during and after pregnancy at a single period, instead of interviewing women at multiple periods. However, this study serves as a foundation for recommending future research on this important issue to explore how IPV victimization before, during and after pregnancy affects the health and well-being of the women of Bangladesh.

## Conclusion and policy implications

The high prevalence rate of IPV and its association with postpartum depression reflect the importance of acknowledging both of them as a significant public health concern in Bangladesh. The strong association between IPV victimization around the time of pregnancy and the likelihood a mother will exhibit PPD symptoms reinforces the need to conduct routine screening during pregnancy to identify women with a history of IPV or those currently experiencing IPV who may at risk of developing PPD. This particular cohort of women likely evidence a variety of additional risk factors that further heighten their risks of PPD and could clearly benefit from a range of help and support offerings. In developing countries like Bangladesh, it is important that well-organised referral pathways and support mechanisms and organizations be put in place before a screening program commences [113]. We propose developing, implementing and evaluating interventions to prevent or reduce all forms of IPV, focus on the physical and mental health care needs of the abused women as well as addressing their social support and welfare needs. The findings from this study also call for significant attention in the planning of health education programs for all staff to assess the risk of IPV and associated PPD and for the implementation of holistic interventions aimed at improving maternal mental health services in South Asian countries, including Bangladesh. More longitudinal studies examining the association between IPV and PPD are warranted to gain a better understanding of the relationship and to identify periods of peak prevalence over the first 12 months postpartum.

## Supporting information

S1 File Data file. (SAV)
